# Clinical perspectives for repairing rotator cuff injuries with multi-tissue regenerative approaches

**DOI:** 10.1016/j.jot.2022.06.004

**Published:** 2022-08-24

**Authors:** Xu Zhang, Dan Wang, Zuyong Wang, Samuel Ka-kin Ling, Patrick Shu-hang Yung, Rocky S. Tuan, Dai Fei Elmer Ker

**Affiliations:** a Institute for Tissue Engineering and Regenerative Medicine, Hong Kong; b School of Biomedical Sciences, Hong Kong; c Ministry of Education Key Laboratory for Regenerative Medicine, School of Biomedical Sciences, Hong Kong; d Department of Orthopaedics and Traumatology, Faculty of Medicine, The Chinese University of Hong Kong, Shatin, Hong Kong; e College of Materials Science and Engineering, Hunan University, Changsha, China; f Center for Neuromusculoskeletal Restorative Medicine, Hong Kong Science Park, Hong Kong

**Keywords:** Musculoskeletal biomaterials, Multi-tissue regeneration, Stem cells, Growth factors, Exosomes, Rotator cuff repair, H&E, Hematoxylin and eosin

## Abstract

**Background:**

In the musculoskeletal system, bone, tendon, and muscle form highly integrated multi-tissue units such as the rotator cuff complex, which facilitates functional and dynamic movement of the shoulder joint. Understanding the intricate interplay among these tissues within clinical, biological, and engineering contexts is vital for addressing challenging issues in treatment of musculoskeletal disorders and injuries.

**Methods:**

A wide-ranging literature search was performed, and findings related to the socioeconomic impact of rotator cuff tears, the structure-function relationship of rotator cuff bone-tendon-muscle units, pathophysiology of injury, current clinical treatments, recent state-of-the-art advances (stem cells, growth factors, and exosomes) as well as their regulatory approval, and future strategies aimed at engineering bone-tendon-muscle musculoskeletal units are outlined.

**Results:**

Rotator cuff injuries are a significant socioeconomic burden on numerous healthcare systems that may be addressed by treating the rotator cuff as a single complex, given its highly integrated structure-function relationship as well as degenerative pathophysiology and limited healing in bone-tendon-muscle musculoskeletal tissues. Current clinical practices for treating rotator cuff injuries, including the use of commercially available devices and evolving trends in surgical management have benefited patients while advances in application of stem/progenitor cells, growth factors, and exosomes hold clinical potential. However, such efforts do not emphasize targeted regeneration of bone-tendon-muscle units. Strategies aimed at regenerating bone-tendon-muscle units are thus expected to address challenging issues in rotator cuff repair.

**Conclusions:**

The rotator cuff is a highly integrated complex of bone-tendon-muscle units that when injured, has severe consequences for patients and healthcare systems. State-of-the-art clinical treatment as well as recent advances have resulted in improved patient outcome and may be further enhanced by engineering bone-tendon-muscle multi-tissue grafts as a potential strategy for rotator cuff injuries.

**Translational Potential of this Article:**

This review aims to bridge clinical, tissue engineering, and biological aspects of rotator cuff repair and propose a novel therapeutic strategy by targeted regeneration of multi-tissue units. The presentation of these wide-ranging and multi-disciplinary concepts are broadly applicable to regenerative medicine applications for musculoskeletal and non-musculoskeletal tissues.

## Abbreviations

ADSCs: Adipose-derived mesenchymal stromal cells
ASES: American Shoulder and Elbow Society
ASCR: Arthroscopic superior capsule reconstruction
BMSCs: Bone marrow-derived mesenchymal stromal cells
ECM: Extracellular matrix
FGF-2: Fibroblast growth factor-2
HCT/P: Human cells, tissues, and cellular and tissue-based product
IGF-1: Insulin-like growth factor-1
MSCs: Mesenchymal stromal cells
PRP: Platelet-rich plasma
PCL: Poly-ε-caprolactone
PDO: Polydioxanone
PEEK: Poly ether–ether ketone
PLGA: Poly (lactic-co-glycolic acid)
PLLA: Poly-L-lactic acid
RTSA: Reverse total shoulder arthroplasty
TGF-ß: Transforming growth factor-ß
UCB-MSCs: Umbilical cord blood-derived mesenchymal stromal cells
USCs: Urine-derived stem/progenitor cells
VEGF: Vascular endothelial growth factor

## 1 Introduction

In the musculoskeletal system, bone, tendon, skeletal muscle and their respective interfaces form a multi-tissue unit, which is responsible for generation and translation of muscle contractile force into body movement. The importance of musculoskeletal multi-tissue units on human health and quality of life cannot be underestimated. In the last decade, musculoskeletal disorders and injuries have increased by approximately 20%, becoming the second-highest contributor to global disability (behind mental disorders) [1].

To address challenges in the repair of injured bone-tendon-muscle units such as rotator cuff tears, *in vitro* studies as well as *in vivo* preclinical and clinical trials must incorporate a wide spectrum of knowledge and perspectives from multidisciplinary fields, including developmental and musculoskeletal biology, wound healing and inflammation, biomaterials, and tissue engineering. Previous examinations on the topic of musculoskeletal multi-tissue grafts has included engineering- and biological-centric perspectives with regard to technological advances in biomaterials and tissue engineering [2], as well as recent concepts in developmental biology and wound healing [3], respectively. In this review, we focus on advancing the notion of using multi-tissue regenerative approaches, such as engineered bone-tendon-muscle grafts, as a reparative or restorative intervention in current clinical practice. First, the significance and socioeconomic impact of rotator cuff injuries is described (Section 2). This is followed by a review of the shoulder joint structure–function biomechanical relationship to emphasize the highly integrated nature of bone-tendon-muscle units within the rotator cuff complex (Section 3). Next, we discuss the pathophysiology of rotator cuff injuries and subsequent limited tissue healing, identifying the bone-tendon-muscle unit as a potential clinical therapeutic target (Section 4). Current clinical practice for rotator cuff injuries (Section 5.1) and the commercially available materials used in treatment (Section 5.2) are described to highlight the evolving trends in surgical management, including clinical applications of grafts/scaffolds, stem/progenitor cells, growth factors, and exosomes, as well as their associated healthcare regulations (Section 5.3). Lastly, we present preclinical and clinical evidence in support of using musculoskeletal multi-tissue units for the treatment of rotator cuff tears (Section 5.4). The clinically relevant knowledge presented here serve as the basis for rational design of future biomaterials and cell-based constructs to provide the next generation of clinically practical and cost-effective strategies and medical devices for the treatment of challenging musculoskeletal injuries and diseases.

## 2 Rotator cuff injuries and socioeconomic impact

The rotator cuff is comprised of the supraspinatus, infraspinatus, subscapularis, and teres minor skeletal muscles as well as their respective tendons, which are attached to the humeral bone. Together, these multi-tissue units stabilize and control functional movement of the shoulder. As such, injury to one or more of these four muscle-tendon tissues can result in pain and high disability for patients, representing a great socioeconomic burden for healthcare systems.

On an individual level, rotator cuff injuries affect both young and old. Typically, tear sizes can range from asymptomatic small partial-thickness tears to massive full-thickness tears that cause severe shoulder dysfunction and pain [4]. In younger patients, rotator cuff tears are often the result of occupational injuries, such as carrying heavy objects or repetitive overhead arm movements during athletic activities. In such cases, patients may experience a considerable loss of workdays, returning to work only after about 5 months post-surgery [5]. Also, such patients may only return to sports after about 7.78 ​± ​3.20 months depending on injury severity and repair technique [6]. While patients of an advanced age may also sustain occupational or exercise-related rotator cuff tears, there is also an increased incidence of rotator cuff injury with increased age due to tissue aging and degeneration [7]. As a result, afflicted individuals experience loss in shoulder range of motion, which dramatically reduces quality-of-life.

In addition to substantial impact on individuals, rotator cuff tears entail a huge socioeconomic burden in numerous regions including Asia, Europe, and the United States. In Japan, it is estimated that rotator cuff tears are present in 20.7% of the population, with increased prevalence as people age [8]. This does not bode well for regions such as Hong Kong, where the proportion of residents projected to be 65 years or older is 19.8% by 2029, which will be expected to result in increased rotator cuff injury incidence [9]. In Italy, for example, the expected hospital cost sustained by the national health care system for rotator cuff procedures alone is projected to cost over 1 billion Euros by 2025 [10]. Furthermore, approximately 65% of such repairs are performed in patients aged below 65 years old, who represent the working population [10]. Socioeconomic issues pertaining to lost work time and disability costs are additionally compounded by the rising incidence of rotator cuff repairs, which has increased from 44 per 100,000 person-years in 1998 to 131 per 100,000 person-years in 2011 in Finland [11]. At present, surgical repair offers a way to alleviate the burden of rotator cuff injury and disease. For example, in the United States, around 250,000 rotator cuff repairs are performed each year, and this has been estimated to save the national healthcare system USD 3.4 billion annually [12]. Thus, rotator cuff injuries are prevalent and pose a significant socioeconomic burden on the healthcare systems of various nations.

Altogether, rotator cuff tears are detrimental to the mobility as well as quality-of-life of patients and pose a heavy socioeconomic burden on national healthcare systems. This highlights knowledge gaps in our understanding of rotator cuff injuries and our abilities to treat them with current clinical techniques. As such, development of innovative tissue engineering and regenerative medicine approaches is needed to improve patient outcomes and reduce the significant burden that rotator cuff injuries pose to society.

## 3 Rotator cuff musculoskeletal units and their structure–function relationship

Although current clinical management such as arthroscopic surgery can improve patient outcome by reducing pain and improving shoulder function, recurrent defect (re-tear) rates post-surgery remain undesirable (21% at 1 – 2 years follow-up) [13] with extremely high rates (91 – 94%) [14,15] for large-to-massive injuries. In order to achieve optimal patient outcomes, an in-depth, comprehensive understanding of the rotator cuff musculoskeletal unit and their structure–function relationship is crucial for the development and design of new therapeutic approaches.

### 3.1 Rotator cuff bone-tendon-muscle multi-tissue unit

**Fig. 1:**
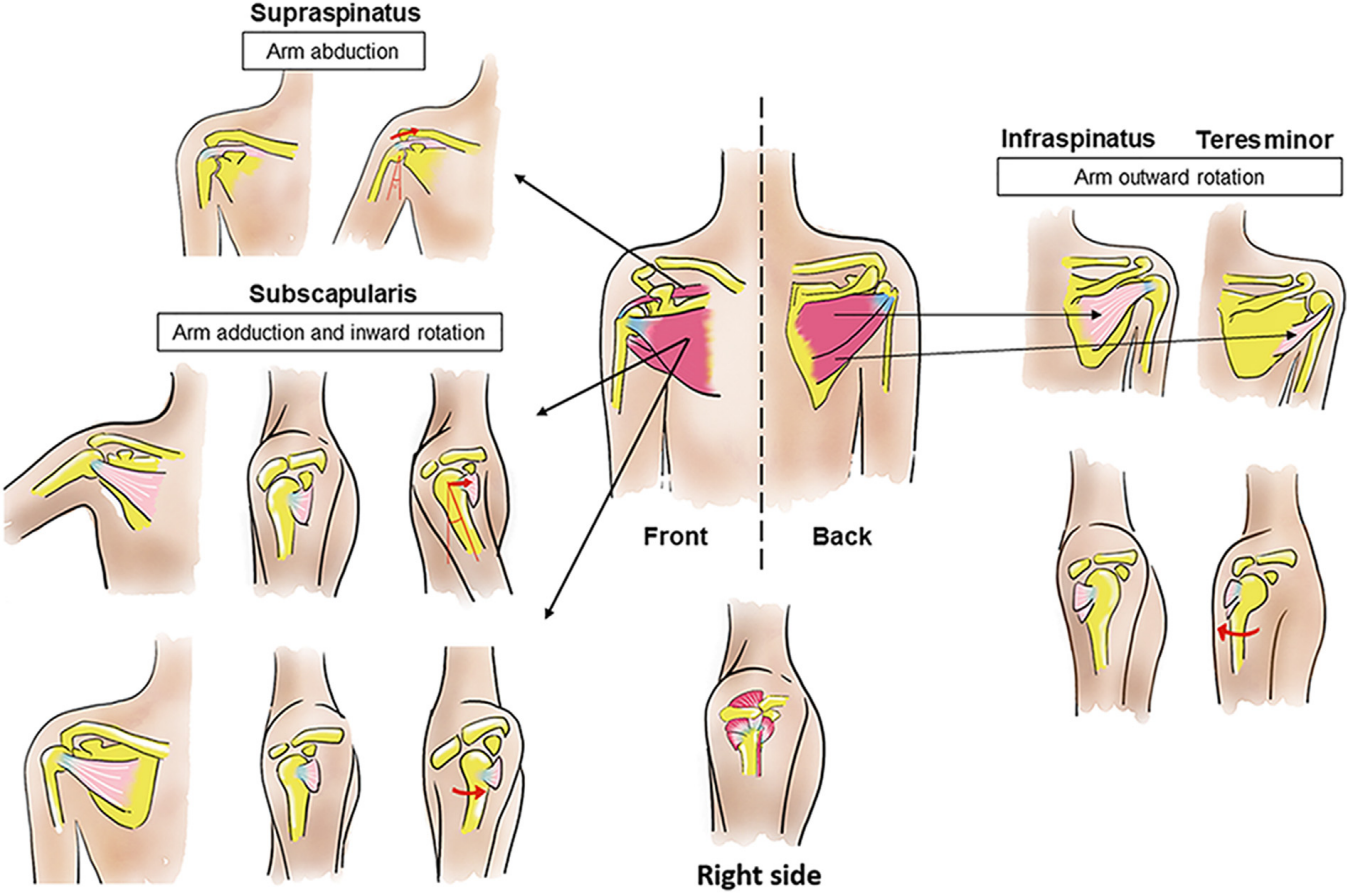
Structure–function relationship of the rotator cuff complex. The supraspinatus muscle-tendon is primarily involved in arm abduction, whereas the subscapularis muscle-tendon predominantly is involved in arm adduction and inward rotation. The infraspinatus and teres minor muscle-tendon are primarily involved in arm outward rotation. Bones, tendons, and muscles are colored yellow, blue, and red, respectively. (For interpretation of the references to color in this figure legend, the reader is referred to the Web version of this article.)

A brief overview of shoulder anatomy, along with the muscular motions facilitated by the rotator cuff, is presented here to depict the structure–function relationship within the rotator cuff. The principal components include musculoskeletal multi-tissue units, such as bone, tendon, ligaments, and skeletal muscle that work in concert to produce a robust range of motion and facilitate joint function (Fig. 1). The skeletal elements of the shoulder consist of the humerus, scapula and clavicle. There are two tuberosities on the humeral head, which are the sites for insertion of shoulder muscles and tendons [[16], [17], [18]]. Apart from the skeletal elements, the shoulder joint is surrounded and covered by a complex network of muscular tissues and their respective tendons including the deltoid, teres major, pectoralis major, latissimus dorsi, biceps brachii, rotator cuff and other supporting muscles [16]. Together, these musculoskeletal elements comprise numerous multi-tissue units that are present in the shoulder.

A bone-tendon-muscle unit is formed by the attachment of different musculoskeletal tissues. For the rotator cuff, the supraspinatus, infraspinatus, subscapularis, and teres minor tendons insert into the humeral head, acting as intermediary elements that connect their respective skeletal muscle to bone. Traditionally, biologists and surgeons have used a reductionist approach to describe the precise location for each of the four rotator cuff tendon footprints. These footprints delineate the site where each tendon inserts into the lesser and greater tuberosity of the humerus and serve as a means to measure tendon loss or act as a guide during surgical repair [19]. However, instead of finding individual muscle-tendon insertion sites, cadaveric studies showed that the rotator cuff tendons actually splay out and interdigitate to form a common and continuous insertion as they approach the humeral head [20]. In addition to interdigitating with one another, rotator cuff tendons also fuse with the articular capsule along its deepest layer before the common insertion into the bone, providing further stability and anchorage [21,22]. Thus, the four bone-tendon-muscle units of the rotator cuff constitute a highly integrated musculoskeletal complex.

### 3.2 Rotator cuff structure–function relationship

Four rotator cuff skeletal muscles and tendons, together with humeral bone are the major force transduction units that drive functional movement of the shoulder. These musculotendinous elements stabilize joints, control the movements and positions of joints, as well as store and release elastic (strain) energy [23]. This enables the shoulder to be one of the most mobile joints in the body and capable of limb flexion (150–180°), extension (40–60°), abduction (150–180°), internal (50–70°) and external rotation (60–90°) [24,25].

These movements are mainly controlled by the rotator cuff, which directly overlays the glenohumeral joint (Fig. 1). The rotator cuff muscles co-contract with the deltoid, compressing the humeral head into the glenoid. Asymmetric contraction of rotator cuff acts to cause humeral head rotation during shoulder motion [16,18,26]. The supraspinatus muscle-tendon unit, which is the most commonly injured rotator cuff tissue, primarily functions to raise the arm above the head, especially within the first 15° of motion [16]. The supraspinatus muscle originates from the supraspinatus fossa and inserts at the superior aspect of the greater tuberosity of the humeral head, with its tendon blending into the articular capsule and infraspinatus tendon below. The subscapularis muscle-tendon unit originates from the subscapular fossa and extends laterally to its insertion on the lesser tuberosity of the humerus. When the arm is not raised, the subscapularis rotator cuff unit acts as an internal rotator whereas when the arm is raised, muscle contraction pulls the arm in a forward and downward motion. Both the infraspinatus and teres minor muscle-tendon units have a similar role. Primarily, the function of these two muscles is to provide the external rotation force that pull the arm away from the body midline, moving the arm backwards [16,27]. Although each rotator cuff muscle-tendon is responsible for particular set(s) of musculoskeletal motion, they do not act in isolation but instead can be influenced by their neighboring muscles due to the highly integrated nature of the rotator cuff muscle-tendon tissues as mentioned in Section 3.1. Rotator cuff muscles and tendons are also additionally assisted by non-rotator cuff musculature and tendons such as the deltoid, teres major, pectoralis major, latissimus dorsi, and the long head of the biceps. Dynamic muscle forces transmitted via tendinous attachments to bone within this complex environment of skeletal elements and ligamentous restraints produce intricate varieties of force couples that account for robust shoulder movements. Injury to one or more of these components through long-term overuse or acute trauma disrupts this complex interrelationship and places the shoulder at increased risk of injury [17,18].

### 3.3 Benefit of understanding structure–function relationship of rotator cuff bone-tendon-muscle units

As such, rotator cuff injuries present numerous pathophysiological changes in multiple locations including bone-tendon interface, tendon, muscle, and bone, which challenge rotator cuff repair. This highlights a structure–function relationship which exists among the four rotator cuff muscle-tendon attachments that translate musculoskeletal force generation into robust shoulder movement. Additional studies that enhance our understanding of the bone-tendon-muscle structure–function relationship may assist the clinical repair of rotator cuff tear to improve the patient outcomes. For example, Liu et al. systematically discussed the suitability of different approaches for modelling aspects of rotator cuff tears and identified suitable assessment methods such as gait analysis for evaluating recovery of shoulder function [28]. Furthermore, a thorough understanding of the disease itself and the healing process is fundamental for clinical treatment, which will be further described in the following section.

## 4 Pathophysiology of rotator cuff injury and healing process

Given that rotator cuff injury is caused by acute trauma, chronic overuse (repetitive stress), or gradual aging (degeneration) of one or more bone-tendon-muscle tissues, understanding rotator cuff pathophysiology is crucial for identifying novel clinical targets for therapeutic development. Since the rotator cuff exists as an integrated multi-tissue complex, injuries are not only confined to the tear site but also to multiple components of the musculoskeletal tissue unit including tendon, skeletal muscle and bone.

### 4.1 Bone–tendon interface or enthesis pathophysiology

Typically, tears primarily occur at the bone-tendon interface or enthesis. This is because there is a severe mechanical mismatch between hard and soft tissues such as bone and tendon/ligament [29]. Although the enthesis is characterized by a graded transition in mineral content, collagen fiber orientation, and biochemical composition that reduces stress concentrations resulting from this mechanical mismatch [30], it is still susceptible to injury due to acute trauma, chronic overuse, or aging. Robust healing response is observed after the injury, but enthesis tissue structure is altered and not restored to its native state [31]. Instead, fibrovascular scar tissue typically forms, instead of regenerated native, graded bone-tendon transition. The absence of fibrocartilaginous insertion at the bone-tendon interface results in stress concentrations; in the presence of mechanically weaker scar tissue, the likelihood for recurrent defects increases [32]. A comprehensive overview of the structural and functional changes that occur at this interface following injury is summarized in the work of Lu et al. [33], Longo et al. [31], Rodeo et al. [34], Rothrauff et al. [30], and Thomopoulos et al. [35].

### 4.2 Tendon degeneration

Although distant from the typical location for rotator cuff tears, tendons are susceptible to degeneration. In full-thickness tears, the torn tendon retracts and its compositional and biomechanical properties typically are not restored to pre-injury levels [32,36,37]. The collagen type ratio within tendon is altered, such that collagen type III is increased and becomes predominant instead of collagen type I. Thinning and disorientation of collagen fibers, myxoid and hyaline degeneration, chondroid metaplasia, calcification, fatty degeneration and vascular proliferation have been reported in degenerative tendons [38]. The presence of vascular proliferation together with fatty degeneration disrupt collagen fiber continuity and can reduce tensile properties [38]. Indeed, the tensile strength in the healed tendons has been reported to be approximately 30% relative to intact tendons [39]. In addition to incomplete bone-tendon healing as well as degenerative changes to tendon, another important obstacle that hinders rotator cuff treatment is progressive muscle atrophy or degeneration, particularly in chronic or “irreparable” rotator cuff tears [40].

### 4.3 Muscle degeneration

Indeed, skeletal muscle pathophysiological changes pose a significant obstacle for recovery. Such atrophic and degenerative changes in skeletal muscle include decreased tissue volume/mass and sarcomere number, increased fibrosis and fatty degeneration, reduced capillary density, and alterations in muscle pennation angle and fiber-type switching [[41], [42], [43], [44], [45], [46], [47], [48]]. These changes are typically correlated with inferior rotator cuff healing as well as poor surgical and non-surgical outcomes [49]. In a clinical study with 38 rotator cuff tear patients, the degree of muscle atrophy and fatty degeneration of the infraspinatus muscle showed a strong negative correlation with functional scores, such as the American Shoulder and Elbow Society (ASES) and Constant scores, in most cases [49]. The primary cause for this may stem from mechanical unloading due to the detachment of the torn tendon although muscle denervation caused by suprascapular nerve injury may also play a contributing role [50].

In addition to muscle fatty degeneration and architectural changes, muscle retraction affects the biomechanical properties of the muscle-tendon unit. The retracted muscle becomes stiffer with decreased elasticity and viability. As such, the impaired mechanical properties of the muscle may explain the loss of shoulder strength following a large-to-massive rotator cuff tear. Indeed, a positive correlation was found between tear size and bundle elastic modulus in torn supraspinatus muscle samples [51]. The degeneration and retraction of both muscle and tendon tissues can alter biomechanical (tension) forces generated within the rotator cuff, resulting in impaired musculoskeletal movements.

Central to this idea is the role of muscle active and passive tension. When tendon and muscle tissues are retracted and/or degenerated, the ability of muscle to generate active tension is impaired. Specifically, loss of muscle mass and decreased length of its contractile elements (sarcomeres) reduces active tension [43]. Indeed, massive rotator cuff tears have been shown to correlate with reduced muscle mass, muscle fiber length, and sarcomere length [43]. In cadaveric-simulated tendon retraction studies, partial detachment (up to two-thirds tendon width) or retraction of the supraspinatus tendon (up to two-thirds tendon width and half its length) only resulted in minor reduction (≤5%) of force transmission from the rotator cuff [52]. However, complete detachment (whole tendon width) or retraction of the supraspinatus tendon (whole tendon width and half its length) resulted in decreased force transmission by 11% and 17%, respectively [52]. In cadaveric, simulated muscle and tendon retraction studies, removal of one-third, two-thirds, and the entire supraspinatus tendon resulted in decreased force transmission by 19%, 36%, and 58%, respectively [52]. Complete repair of these simulated defects restored force transmission to levels similar to that of intact rotator cuff [52]. While it is generally recognized that some tension within the rotator cuff is necessary for optimal function, excessive passive tension following repair is correlated with inferior patient outcomes, including decreased strength and increased pain [53]. While the reasons remain to be determined and are likely multi-factorial, it has been suggested that repairing retracted muscle and tendon tissues back to the native rotator cuff footprint may damage muscle by overstretching its sarcomeric contractile elements [43], thus further decreasing muscle efficiency and active tension [54].

The loss of these tissues not only affects active tension but can also affect passive tension. Since passive muscle tension (transmitted via the rotator cuff and biceps tendon) compresses the humeral head into the glenoid cavity [17], loss of superior rotator cuff tendons such as the supraspinatus can result in the humeral head migrating out of the “socket” and upwards [[55], [56], [57]]. The displacement of humeral head narrows the subacromial space and hinders tendon gliding, resulting in inflammation and subacromial or shoulder impingement syndrome [17,58]. Together, these studies highlight the importance of restoring rotator cuff tissue tension to ensure proper musculoskeletal anatomy for efficient force generation and transmission.

### 4.4 Bone loss

Besides changes in tendon and muscle, bone loss has also been observed in chronic rotator cuff injury. In rat models with supraspinatus tendon transection, bone density in the greater tuberosity was markedly decreased in the delayed repair group compared to those with immediate repair [59]. Consistent with animal studies, significantly greater osteopenic changes in the greater tuberosity were observed in patients with chronic retracted rotator cuff tears, compared to those with acute minimally retracted tears [60]. This decreased bone quality may complicate surgical repair of the rotator cuff and exacerbate already inferior tendon-to-bone healing [59].

### 4.5 Benefit of understanding multi-tissue degeneration for clinical treatment

In summary, rotator cuff tears often occur at the bone-tendon interface. However, degenerative changes to associated bone, tendon, and skeletal muscle tissues also occur. Thus, additional attention on the bone-tendon-muscle unit, as opposed to only restoring the torn tendon back to the humeral bone, should benefit clinical treatment of rotator cuff tears by giving broader consideration to the pathophysiological degenerative changes that occur as a result of disease and injury.

## 5 The need for new clinical targets in the treatment of rotator cuff injuries

### 5.1 Current clinical practice for rotator cuff injuries

Current clinical practice for rotator cuff injury treatment target issues caused by pathophysiological degeneration. Such symptoms of rotator cuff tears include pain, loss of strength, limited shoulder motion and instability of the shoulder joint. Treatments are broadly divided into non-surgical managements and surgical repairs, which are indicated according to clinical factors, such as age, tendon location, mode of injury, and size of defect [58,61,62].

#### 5.1.1 Non-surgical management

Non-surgical management includes rest, physical therapy, and medications (anti-inflammatory drugs or corticosteroid injection) coupled with non-invasive monitoring methods (ultrasound and shoulder examinations). Patients with tendonitis, partial thickness tears, small (<1–1.5 ​cm) full-thickness tears should be considered for initial conservative treatment due to the reduced risk for irreversible, chronic rotator cuff changes. Non-operative treatment at the initial stage may also be beneficial for aged patients (> 65 years) who have low physical demands and are afflicted with chronic full-thickness tears or large irreparable tears with chronic, irreversible muscle damage [61,63,64]. However, non-operative treatments are still associated with a number of risks that include lack of spontaneous healing, tear progression, muscle fatty degeneration and retraction, tendon retraction, increased difficulty of tendon mobilization, and development of arthritis in the long-term [61,[63], [64], [65]]. Therefore, early surgical repair is strongly recommended for individuals with acute tears and in younger patients (< 65 years old) with chronic, reparable tears or substantial tears (> 1 ​cm) without significant chronic muscle changes [61,[63], [64], [65]].

#### 5.1.2 Surgical management

**Fig. 2:**
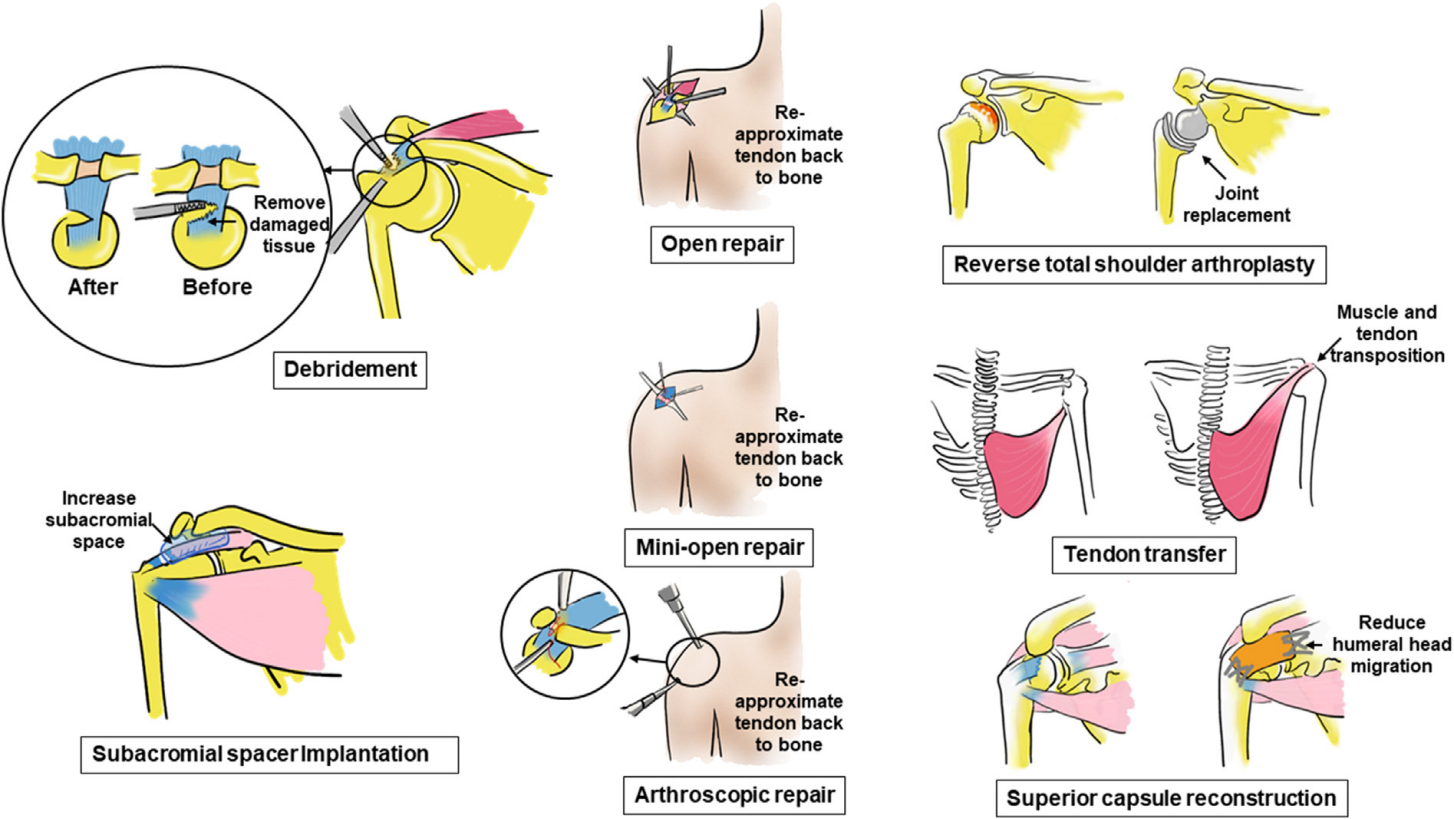
Different surgical options for rotator cuff injuries. These operative procedures include debridement, open repair, mini-open repair, arthroscopic repair, reverse total shoulder arthroplasty, tendon transfer, superior capsule reconstruction, and subacromial spacer implantation. Bones, tendons, and muscles are colored yellow, blue, and red, respectively. (For interpretation of the references to color in this figure legend, the reader is referred to the Web version of this article.)

Surgical management for rotator cuff tears can range from simple debridement to complex procedures such as reverse total shoulder arthroplasty (RTSA), tendon transfer, and implantation of tissue engineered scaffolds or grafts (Fig. 2).

Tissue debridement involves removal of loose fragments of tendon, thickened bursa, and other damaged tissue to relieve pain, whereas subacromial spacer implantation involves positioning a biodegradable, fluid-containing balloon between the acromion and humeral head to permit smooth gliding and restoration of shoulder function.

Open repair, mini-open repair, and arthroscopic repair are repair methods that seek to anatomically affix tendon back to bone via combinatorial application of suture anchor(s) and sutures. While patient outcome for these methods have largely been satisfactory, a systematic review of studies from 2003 to 2014 reported that the pooled rate for recurrent defects was as high as 79% for arthroscopic repair of chronic massive rotator cuff tears [66]. As such, other surgical methods that do not involve anatomic repair have been developed.

In contrast to anatomic repair of tendon back to bone, several reconstructive methods have been developed which restore shoulder function in a non-anatomic manner. These reconstructive procedures include: RTSA, tendon transfer, and arthroscopic superior capsule reconstruction (ASCR).

RTSA reconstructs the shoulder joint of elderly patients using a ball and socket implant affixed to the humerus and scapula, respectively, which is in reverse relative to native shoulder anatomy. RTSA relies on the deltoid muscle, instead of the rotator cuff, to power and position the arm. However, RTSA is associated with a high complication rate. The reported overall complication rate of primary RTSA is approximately 15% due to instability, infection, hematoma, scapular notching, glenoid loosening, neurological injury, heterotopic ossification, acromial and scapular spine fractures, intraoperative fractures, and component disengagement [[67], [68], [69]].

Tendon transfer is another reconstructive option for treating irreparable massive rotator cuff tears. This procedure helps to restore shoulder mechanics and allow patients to reduce pain and regain partial shoulder function [70]. This is performed by utilizing an existing, healthy muscle-tendon unit to substitute for the function of the injured rotator cuff muscle-tendon unit. In this procedure, the transferred tendon's insertion site is changed to redistribute skeletal muscle contractile force to power the injured rotator cuff but the transferred tendon's origin remains unaltered, resulting in a non-anatomic procedure. The benefit of tendon transfers is that they are not limited by the quality of the remaining rotator cuff tendon and muscles, but instead provide a functionally contracting muscle-tendon unit in comparison to other passive techniques such as subacromial spacer implantation. A variety of tendon transfer options (pectoralis major, latissimus dorsi, and trapezius tendon) are available for the treatment of patients with a massive irreparable rotator cuff tear. Good-to-excellent results have been reported in short to intermediate-term and long-term clinical studies for latissimus dorsi transfer to treat irreparable posterosuperior rotator cuff tears [[71], [72], [73], [74]]. However, several complications exist. These include the postoperative formation of hematoma, infection, late/secondary tendon rupture, anchor pull-out, and nerve palsies [75]. Recent surgical advancements such as transfer of trapezius tendon have been described, with advantages derived from the procedure's technical ease and ability to produce a muscle-tendon unit whose line of pull, tension, and excursion forces are similar to the infraspinatus rotator cuff unit [76]. However, more extensive clinical studies examining patient outcome at mid-to-long term follow up are required. Thus, tendon transfers remain a viable strategy for restoring shoulder function.

ASCR is a relatively new surgical method developed by Mihata et al. which affixes a fascia lata autograft from the superior glenoid onto the greater tuberosity of the humeral head to restore shoulder joint stability [77]. ASCR is gaining popularity and is considered a reliable and useful alternative for irreparable rotator cuff tears [78]. The reported short-term outcomes (minimum follow-up of two years) are promising with one study reporting that in 23 patients, the ASES score increased from 23.5 to 92.9 and the acromiohumeral distance also significantly increased by 4.1 ​mm after surgery [78]. Recently, a 5-year follow-up study of ASCR showed an increase of 63.3 points for the ASES score, 39.9 points for the JOA score, 66° for active elevation, and 4.7 ​mm for acromiohumeral distance compared with the preoperative values, with only 3 out of 30 patients having a graft tear [79]. However, a high rate of graft tear (9 out of 31 patients) as assessed by MRI was reported after an average of 12.8 months following ASCR [80]. Thus, studies with longer-term follow-up and larger cohort of patients are needed.

#### 5.1.3 Evolving trends in surgical management: use of biomaterials to supplement rotator cuff repair or reconstruction

In spite of the unparalleled success of surgical repair and reconstructive techniques for improving patient outcome, it can still be difficult to regain full range of shoulder motion. As such, commercially available biomaterials such as rotator cuff patches and synthetic scaffolds have been introduced with these techniques.

In arthroscopic repair, it can be challenging to re-attach the torn tendon back to bone for large-to-massive rotator cuff tears [14]. As such, surgeons have devised new methods that utilize native tissues, biological patches or synthetic scaffolds as either augmentation or interpositional grafts for rotator cuff repair. In a recent clinical trial, GraftJacket™ (acellular human dermal matrix) of proper size and tissue-matched thickness was used as an interpositional allograft to repair the tendon back to the footprint. A statistically significant improvement in Oxford shoulder scores (from a mean of 22–45.5) over a follow up of 18 months was demonstrated and host tissue integration was observed in one patient with arthroscopy [81]. However, the long-term outcomes and safety for these kinds of rotator cuff repair are unclear, and largely depends on the patient etiology and graft characteristics.

For ASCR, donor tissue such as fascia lata is used to treat irreparable, large-to-massive rotator cuff tears. To avoid donor-site morbidity stemming from fascia lata harvest, human acellular dermal patch allograft (ArthroFlex®), which has shown promising results in patch-augmented rotator cuff repair, has also been used for superior capsule reconstruction [82,83]. Recently, in a one-year follow up study involving 59 patients undergoing ASCR with dermal allograft, around 70% of cases showed promising outcome with the ASES scores improved from 43.6 to 77.5, and the subjective shoulder value score improved from 35.0 to 76.3 [84]. Such studies, while promising, will require longer-term follow up of patients.

### 5.2 Commercially available biomaterials used in treatment of rotator cuff tears

In the last few decades, several new tissue-engineered materials, including naturally derived biomaterials and synthetic polymers, have been used in the development of new tendon substitutes and other medical devices (suture anchor) for rotator cuff injury [85].

#### 5.2.1 Naturally derived tendon scaffolds

**Fig. 3:**
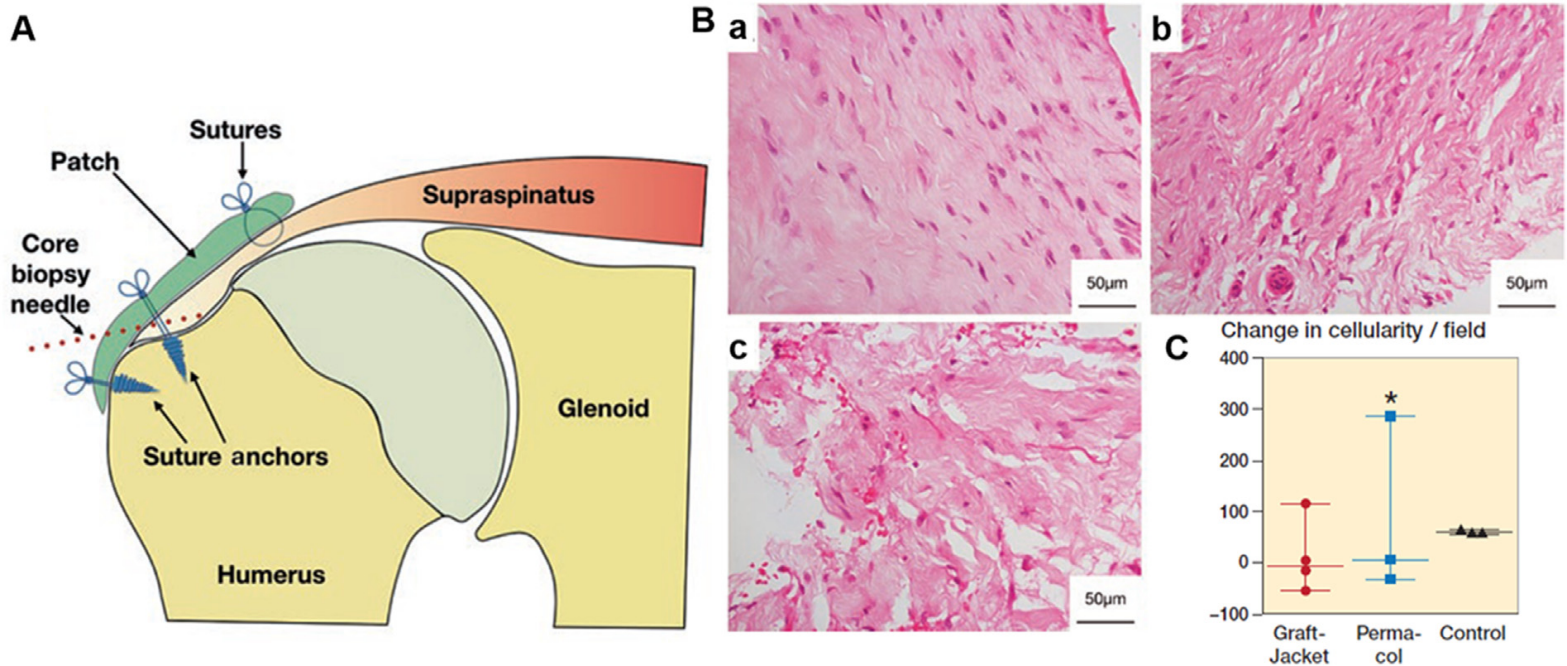
Changes in cellularity following standard rotator cuff repair or patch-augmented rotator cuff repair. (**A**) Schematic showing the biopsy sample site (Core biopsy needle, dotted line) at 4-week post-surgical repair. (**B**) Representative hematoxylin and eosin (H&E) histological staining showed cellularity changes following patch augmentation or standard repair (no patch; control) group. H&E staining was performed on (**a**) control, (**b**) GraftJacket™, and (**c**) Permacol™ groups, with patch augmentation showing increased disruption of the tendon extracellular matrix. (**C**) Histological quantification showed lower cellularity per field of view for patch-augmented repair (GraftJacket™ and Permacol™) relative to standard repair control. ∗ denotes a patient who received Permacol™ and exhibited a grossly different tissue response. Copyright obtained from Taylor & Francis and adapted from Mustafa S Rashid et al., 2020.

Naturally derived tendon scaffolds for rotator cuff repair are derived from the natural extracellular matrix (ECM) of various human (autograft or allograft) and animal tissues, (xenograft) including dermis, small intestine submucosa, fascia lata, and pericardium. The ECM materials are processed via proprietary methods prior to being marketed as patches for rotator cuff repair. Typically, such processing involves decellularization, chemical cross-linking, lamination of multiple layers, and/or lyophilization. Popular, commercially available, naturally derived scaffolds include GraftJacket™, AlloPatch®, ArthroFLEX®, TissueMend®, Bio-Blanket®, Zimmer®/Permacol™, Conexa™, Biotape®, Restore™, CuffPatch®, Tutopatch® and OrthADAPT® (see Table 1) [86]. Due to their natural origin, such scaffolds often exhibit well-defined three-dimensional microstructure and natural porosity, which not only provide temporary mechanical support but also facilitate host cell attachment, proliferation, and migration. The incorporation and subsequent remodelling of the scaffold by host cells may enhance and accelerate the native tissue repair. However, a major concern for most commercially available grafts is their low mechanical strength. Typically, their linear modulus and stiffness are an order of magnitude lower compared to native rotator cuff tissues [86,87]. Also, although naturally derived scaffolds are often touted for their biological activity and biocompatibility, recent histological and immunohistochemical studies have indicated a lack of patient benefit with regard to increased cellularity or vascularity after rotator cuff patch augmentation relative to standard repair control (Fig. 3) [88]. Other issues arising from use of naturally derived tendon scaffolds include ill-defined degradation rate due to the variable nature of the source material(s) [86,89]. Such issues may be addressed by slight modifications to the surgical technique [81,90,91]. For example, doubling the GraftJacket™ sheet to match patient tissue thickness (as an interpositional graft) resulted in improved Oxford shoulder scores for 18 patients with massive rotator cuff tears over an 18-month duration [81].

**Table 1:** Commercially available scaffolds for rotator cuff repair.

Brand name	Composition	Company	Study type/Level of evidence	Sample size	Repair technique	Follow-up period	Clinical outcome	Ref.
Naturally derived								
GraftJacket™	Human dermis	Wright Medical, Arlington, TN, USA	Cohort/3	47	Interposition	9.1 years	Improved clinical outcomes (Oxford shoulder score) and high patient satisfaction that was maintained at 9.1 years.	Modi et al., 2022 [189]
AlloPatch®	Human dermis	MTF Sports Medicine, Edison, NJ, USA	Retrospective case series/4	14	Augmentation	1 year	Majority of rotator cuffs (85.7% were intact) with improved clinical outcomes (Constant score, Flexilevel Scale of Shoulder Function, pain score, scapular plane abduction, and strength).	Agrawal, 2012 [190]
ArthroFLEX®	Human dermis	Arthrex, LifeNet Health, Virginia Beach, VA, USA	Controlled laboratory study (Cadaver)/3	25	Interposition or Augmentation	—	Both augmentation and interposition repair increased ultimate load in cadaver shoulders.	Beitzel et al., 2022 [191]
TissueMend®	Fetal bovine dermis	Stryker Orthopedics, Mahwah, NJ, USA	Technical note/6	NA	Augmentation	NA	NA	Seldes et al., 2006 [192]
Bio-Blanket®	Bovine dermis	Kensey Nash Corporation, Exton, PA, USA	Review/7	NA	Augmentation	NA	NA	Coons and Barber, 2006 [193]
Zimmer or Permacol™	Porcine dermis	Medtronics, Mansfield, MA, USA	Case series/4	10	Augmentation	4 weeks	Disruption of extracellular matrix underlying both patches was observed. 1 patient had adverse tissue immune response.	Rashid et al., 2020 [88]
Conexa™	Porcine dermis	Tornier, Edina, MN, USA	Case series/4	4	Interposition	10.5 months	Favourable remodeling of graft with vessel infiltration without evidence of inflammation, foreign body reaction, or tissue rejection.	Christian et al., 2021 [194]
Biotape®	Porcine dermis	Wright Medical, Arlington, TN, USA	Review/7	NA	Augmentation	NA	NA	Karuppaiah et al., 2019 [195]
				24	NA	About 2 weeks	Comparable proliferation and tendon gene expression as other commercial grafts.	Smith et al., 2016 [196]
Restore™	Porcine small intestine submucosa	DePuy Orthopedics, Warsaw, IN, USA	Controlled trial/3	31	Augmentation	2 years	Poor clinical outcomes including lower strength and more impingement in external rotation with slower resolution of pain and more difficulty in daily living activities.	Walton et al., 2007 [197]
CuffPatch®	Porcine small intestine submucosa	Arthrotek, Warsaw, IN, USA	Controlled laboratory study (Rat)/3	126	Interposition	112 days	Presence of foreign-body giant cells, chronic inflammation and accumulation of dense, poorly organize fibrous tissue.	Valentin et al., 2006 [198]
Tutopatch®	Bovine pericardium	Tuto-gen Medical GmbH, Neunkirchen am Brand, Germany	Retrospective case series/4	152	Augmentation	3 years	Similar clinical outcome (UCLA score), retear rate, pain, strength, and elevation as control (open repair) group.	Ciampi et al., 2014 [93]
OrthADAPT®	Equine pericardium	Pegasus Biologic Inc., Irvine, CA, USA	Controlled laboratory study (Rat)/3	41	NA	3 months	Increased maximum load compared to suture only repair at 3 months.	Tornero-Esteban et al., 2015 [199]
**Synthetic (Non-degradable)**								
Leeds-Keio®	Polyester ethylene terephthalate	Xiros plc, Neoligaments, Leeds, UK	Randomized controlled trial/1	39	Augmentation	2 years	Improved clinical outcomes (Hospital for Special Surgery score), decreased pain, increased range of motion, and increased strength.	Tanaka ​et al., 2006 [200]
Poly-tape®	Polyester ethylene terephthalate	Yufu Itonaga Co., Ltd, Tokyo, Japan	Controlled laboratory study (*in vitro*)/3	24	NA	About 2 weeks	Comparable proliferation and tendon gene expression as other commercial grafts.	Smith et al., 2016 [196]
Mersilene® mesh	Polyester ethylene terephthalate	Ethicon, Inc., Somerville, NJ	Case series/4	41	Interposition	43 months	Improved clinical outcomes (Constant score), reduced pain and improved daily living activities.	Audenaert et al., 2006 [201]
Lars® ligament	Terephthalic polyethylene polyester	Ligament Augmentation and Reconstruction System, Dijon, France	Controlled laboratory study (*in vitro*)/3	24	NA	About 2 weeks	Comparable proliferation and tendon gene expression as other commercial grafts.	Smith et al., 2016 [196]
Gore-Tex® patch	Polytetrafluoroethylene	Gore and Associates, Flagstaff, AZ, USA	Case series/4	28	NA	44 months	Improved clinical outcome (JOA score), reduced pain, and increased abduction strength.	Hirooka et al., 2002 [202]
Marlex®	High-density polyethylene	C.R.Bard, Mullayhill, NJ, USA	Case series/4	9	Capsular reconstruction	3–48 months	Improved clinical outcome with respect to joint instability although there were two surgical complications.	Gortzak et al., 2010 [203]
Repol Angimesh®	Polypropylene	ANGIOLOGICA BM Srl, Pavia, Italy	Retrospective case series/4	152	Augmentation	3 years	Improved clinical outcome (UCLA score) as well as, lower retear rate, lower pain, increased strength, and increased elevation as control (open repair) group.	Ciampi et al., 2014 [93]
**Synthetic (Degradable)**								
X-Repair	Poly-L-lactic-acid	Synthasome Inc., San Diego, CA, USA	Case series/4	18	Augmentation	42 months	Improved clinical outcomes (ASES score) with 78% intact repair at 42 months.	Proctor, 2014 [204]
Artelon® or SportMesh™	Polyurethane urea polymer	Artimplant AB, Sweden	Case study/6	3	Augmentation	6 months-2.5 years	Improved clinical outcomes (WORC and Oxford score) with integrity of rotator cuff retained at 15-months postoperatively for one patient. Remaining two patients showed improved Constant score (17% and 79%) at six months.	Zhaeentan et al., 2011 [205]
Integraft™	Carbon fibre	Hexcel Medical, Dublin, CA	Review/7	NA	Augmentation	NA	NA	Karuppaiah et al., 2019 [195]
BioFiber™	Poly(4-hydroxybutyrate)	Tornier, Edina, MN, USA	Controlled laboratory study (*in vitro*)/3	24	NA	About 2 weeks	Comparable proliferation and tendon gene expression as other commercial grafts.	Smith et al., 2016 [196]

∗NA, not available

#### 5.2.2 Synthetic tendon scaffolds

**Fig. 4:**
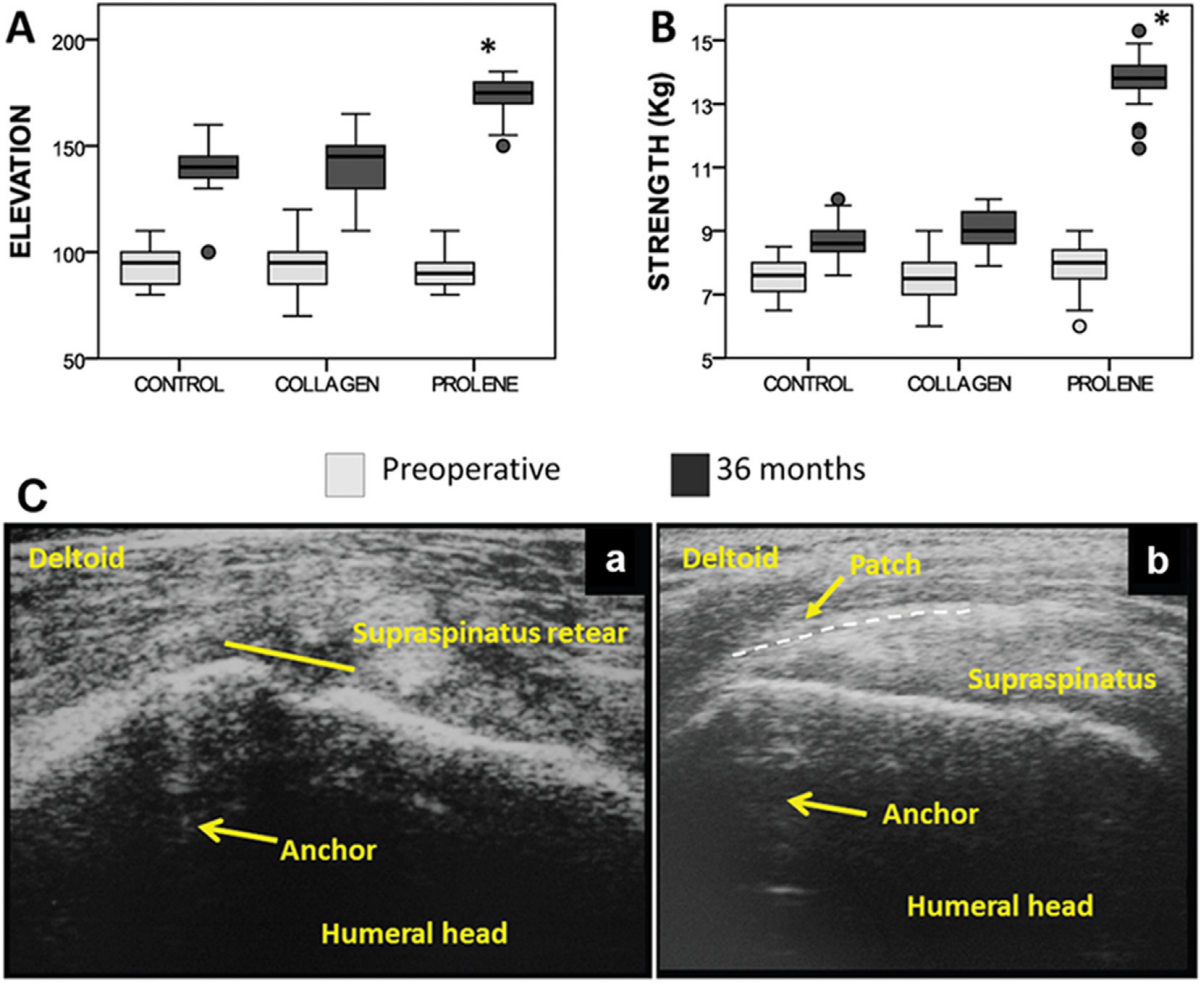
Shoulder function and tissue healing following open repair (control), collagen-mediated rotator cuff repair, and polypropylene (prolene) (Repol Angimesh®, Angiological BM Srl, Pavia, Italy) -mediated rotator cuff repair. (**A**) Boxplots summarizing elevation on the scapular plane; (**B**) abduction strength assessed pre- and 36 months post-operatively. ∗, Statistically significant difference at 36 months between the polypropylene group with other groups. Whiskers indicate minimum and maximum values. Round dots indicate outliers, defined as values higher than 1.5-fold the interquartile range. (**C**) Ultrasound images taken 12 months post-surgery showed (**a**) recurrent supraspinatus defect and collagen patch absorption, and (**b**) maintenance of rotator cuff integrity and the presence of a polypropylene patch. Copyright obtained from SAGE journals and adapted from Pietro Ciampi et al., 2014.

Synthetic tendon scaffolds can be further divided into non-degradable and degradable scaffolds. Non-degradable synthetic scaffolds are based on polymers such as polyester, polyurethane, polypropylene, and expanded polytetrafluoroethylene (Teflon), and can be found in commercially available products such as Leeds-Keio®, Poly-tape®, Mersilene® mesh, Lars® ligament, Gore-Tex®/Teflon™ patch, Marlex® and Repol Angimesh® (see Table 1) [86]. Recent concerns associated with these non-degradable implants include persistent infections, loss of integrity, and long-term safety [92]. As such, there has been a shift towards synthetic degradable scaffolds in recent years, which aims to provide non-permanent support to avoid undesired foreign body reactions as well as accommodate tissue healing and remodelling. Degradable scaffolds can be made of a wide variety of polymers including poly-L-lactic acid (PLLA), poly (lactic-co-glycolic acid) (PLGA), poly-ε-caprolactone (PCL), polydioxanone (PDO), and polyurethane urea, and can be found in commercially available products such as X-repair, Artelon®/SportMesh™, Intergraft™ and BioFiber™ (Table 1) [86,87]. Compared to naturally derived scaffolds, synthetic scaffolds exhibit higher consistency and superior mechanical strength. For example, use of a polypropylene scaffold resulted in increased patient arm elevation (174.71° ​± ​8.18°), strength (13.79 ​± ​0.64 ​kg), and reduced the rate of recurrent defects (17%) relative to control (elevation: 140.68° ​± ​9.84°; strength: 8.73 ​± ​0.54 ​kg; and recurrent defect rate: 41%) and collagen (elevation: 140.61° ​± ​12.48°; strength: 9.03 ​± ​0.60 ​kg; and recurrent defect rate: 51%) groups (Fig. 4) [93]. However, synthetic scaffolds have been traditionally associated with little-to-no bioactivity and poor biocompatibility [89].

Despite the success of both commercially available naturally derived and synthetic scaffolds in rotator cuff patient care, a present limitation of existing tendon substitutes is that they lack features to mimic the bone-tendon interface, which is the most frequent injury site [86]. Thus, the ideal scaffolds for rotator cuff repair should possess superior bioactivity, biocompatibility, and mechanical attributes with the capability to address regeneration of the bone-tendon interface. Within a clinical setting, the current state-of-the-art is to utilize suture anchors for affixing single tissue-mimicking, tendon substitutes to bone.

#### 5.2.3 Suture anchors

As critical elements used during rotator cuff repair, suture anchors have gone through several modifications as clinical practice transitioned from open to arthroscopic repair. Initially, metallic suture anchors with non-absorbable sutures were used, but complications of loosening, migration, articular surface damage, and artifact production in postoperative MRI were reported in numerous studies [[94], [95], [96], [97]]. In response to this, bioabsorbable anchors were developed [96]. However, other complications stemming from rapid degradation can result in loose foreign bodies (suture or non-resorbed parts of the suture anchor) within the glenohumeral space [98], necessitating revision surgery. Recent developments such as new suture anchor designs such as knotless anchor systems [99], use of osteoconductive, biocomposite suture anchors with a slower degradation profile [100], non-degradable, non-metallic suture anchors such as poly ether–ether ketone (PEEK) [101], and suture anchors comprised entirely of suture material [102] have been implemented to address these concerns with good short-term results but long-term clinical outcomes remain to be determined.

In summary, various commercially available biomaterials are used as tendon substitutes and in suture anchors for rotator cuff repair. Further development of such materials is expected to improve the clinical efficacy of rotator cuff repair and aid in the evolution and subsequent refinement of new clinical techniques.

### 5.3 Clinical application of stem/progenitor cells, growth factors, and exosomes

Although there have been numerous advances and developments to existing clinical techniques and commercially available biomaterials, long-term functional recovery for severely injured rotator cuff tissues remains challenging [30,103]. Also, there has been increased recognition that a highly regenerative environment is necessary to ensure complete rotator cuff tissue healing and restoration of shoulder function. As such, there have been preclinical studies and clinical trials involving application of stem/progenitor cells and growth factors for rotator cuff repair and regeneration. Clinical studies examining the benefit of scaffolds and augmentation at the healing tendon-bone interface have been well summarized in Table 1 of Rothrauff et al. [30]. The applications of stem/progenitor cells, growth factors, and exosomes along with their associated regulatory environment are further elaborated in Table 2 and the following sections.

**Table 2:** Clinical studies using stem/progenitor cells, growth factors or their combination for rotator cuff repair.

Cell type/Growth factor	Study type/Level of evidence	Tear size	Sample size	Method of delivery	Follow-up period	Clinical outcome	Ref.
Stem/Progenitor Cells							
BMSCs	Cohort/3	Full-thickness	124 (57/67)	Drilling into the bone marrow was performed in the greater tuberosity.	At a minimum of 2 years	No significant difference in pain, range of motion, strength, overall satisfaction and functional scores. The retear rate was significantly lower.	Jo et al., 2013 [120]
BMSCs	Case-controlled/4	Tear size from 1.5 to 3.5 ​cm	90 (45/45)	BMSCs were injected into the tendon-bone interface.	At a minimum of 10 years	Enhanced healing rate, improved quality of the repaired surface, reduced number of recurrent defects.	Hernigou et al., 2014 [121]
BMSCs	Retrospective cohort/3	Full-thickness	111 (67/44)	Drilling into the bone marrow was performed in the greater tuberosity.	2–24 months	Improved cuff repair integrity and lower retear rate in large-massive tears.	Taniguchi et al., 2015 [206]
ADSCs	Cohort/3	Full-thickness	70 (35/35)	Injection of adipose-derived MSCs loaded in fibrin glue during rotator cuff repair.	28.3 ​± ​3.8 months	No clinical differences in the 28-month period of follow-up compared to the conventional group.	Kim et al., 2017 [122]
Growth Factors							
PRP	Randomized controlled trial/1	Subacromial impingement syndrome/partial-thickness	60 (30/30)	Injection of autologous PRP into the subacromial bursa.	At a minimum of 2 years	No improvement for clinical outcomes. May have potential deleterious effects on healing tendons.	Carr et al., 2015 [153]
PRP	Randomized controlled trial/1	Medium-sized to large cuff tear	102 (52/50)	Delivery of autologous PRP over the cuff surface through the arthroscopic portal.	At a minimum of 2 years	Visual analog scale scores were lower at 1, 3 and 6 months; Constant-Murley scores improves at 12 and 24 months; UCLA acores were higher at 6 and 12 months; Retear rate decreased at 24 months for large tears.	Pandey et al., 2016 [152]
PRP	Randomized controlled trial/1	Complete rotator cuff tear	120 (60/60)	Intraoperative pure PRP injection.	6 and 24 months	No significantly improved function at 3, 6, and 24 months.	Flury et al., 2016 [207]
PRP	Randomized controlled trial/1	Full-thickness	60 (30/30)	Injection of autologous PRP.	12 months	Lower recurrence rates.	Zhang et al., 2016 [151]
PRP	Randomized controlled trial/2	Complete supraspinatus tear	51 (26/25)	Liquid PRP prepared by apheresis with autologous thrombin was applied in the tendon-to-bone interface.	60 months	No improvement for clinical or structural results at 60-month follow-up.	Malavolta et al., 2018 [148]
LR-PRP	Prospective randomized therapeutic trial/2	Rotator cuff tear	87	Ultrasound guided injection of leukocyte-rich PRP.	12 months	No improvement by patient-reported outcome measures and Constant score at 1 year postoperatively.	Snow et al., 2020 [147]
rhBMP-12	Randomized controlled trial/2	Full-thickness (2–4 ​cm wide)	20 (16/4)	rhBMP-12/absorbable collagen sponge (ACS) was applied to the footprint.	12 months	Functional recovery in theVAS score for pain,ROM, and isometric strength was similar compared to the control group	Ide et al., 2017 [138]
**Combination of Stem/Progenitor Cells and Growth Factors**							
Subacromial bursa, cBMA, PRP	Therapeutic case series/4	Tears with at least 2 tendons	16	Arthroscopic rotator cuff repair augmented using subacromial bursa, cBMA, and platelet-rich plasma delivered to the injury site.	12.6 ​± ​1.8 months (range 12–19 months)	Improvement in ASES, Constant-Murley, SANE and pain scores.	Muench et al., 2020 [208]

#### 5.3.1 Application of stem/progenitor cells

Stem/progenitor cells are characterized by their self-renewal capacity and potential to differentiate into one or more cell type(s). The main aim of using stem/progenitor cell therapy in surgical rotator cuff repair is to either replace injured cells via differentiation into musculoskeletal cells, encourage tissue regeneration via their paracrine actions, or a combination of both. The most widely studied cells within the context of rotator cuff injury treatment are the multipotent adult mesenchymal stromal cells (MSCs), which can be obtained from bone marrow [104], adipose tissues [105], muscle [106], tendon [107,108], synovium [109], periosteum [110], umbilical cord [111,112], peripheral blood [113] and urine [114]. Traditionally, bone marrow-derived MSCs (BMSCs) have been most commonly reported although recent promising studies have focused on other MSCs derived from adipose, tendon, synovium and umbilical cord blood.

For BMSC-mediated regeneration, there have been a large number of preclinical studies and clinical trials. In preclinical studies, efforts have been focused primarily on delivery of BMSCs alone, BMSCs in combination with stimulating factors such as platelet-rich plasma [115], BMSCs in combination with tissue engineered scaffolds/grafts such as demineralized bone matrix [116], BMSCs genetically modified with membrane type 1 matrix metalloproteinase [117], or using BMSC-derived exosomes as a cell-free product [118]. The details of these preclinical studies are well summarized by Xu et al. [119]. In clinical studies, early efforts have focused on endogenous recruitment of BMSCs with promising results. The first clinical study to investigate the impact of BMSCs was reported by Jo et al. [120]. BMSCs were recruited from patients endogenously using a “multiple channeling” procedure, which as the name suggests, involved the creation of multiple channels in the greater tuberosity during the surgical repair of full-thickness rotator cuff tear. Patients were followed for 2 years and although clinical outcomes including pain, range of motion, strength, overall satisfaction and functional scores were not statistically different, the recurrent defect rate of the multiple channeling group (22%) was significantly lower than that of the conventional group (45.2%) [120]. This improved outcome was attributed to increased recruitment of endogenous MSCs from the proximal humerus to the wound site [120] but actual quantification of the BMSC numbers was not performed. To address this, Hernigou et al. performed a study that evaluated the efficiency of biologic augmentation of rotator cuff repair via exogeneous injection of iliac crest-derived BMSCs (51,000 ​± ​25,000) to the injury site. Similarly, the authors reported enhanced healing and improved the quality of the repaired surface as determined by ultrasound and MRI in the BMSCs injection group [121]. Taken together, these findings suggest that BMSC-mediated regeneration is promising for rotator cuff repair.

In addition to the BMSCs, other promising candidate cell types for rotator cuff regeneration include adult-derived adipose, tendon, synovium, peripheral blood and urine-derived MSCs. Adipose tissue is a highly practical source of MSCs as large quantities of MSCs can be easily obtained without the need for culture and expansion. Adipose-derived, mesenchymal stromal cells (ADSCs) have shown good potential in augmenting rotator cuff repair in animal models and are a promising avenue for future research. Kim et al. reported a clinical study that assessed the effect of exogenously injected ADSCs together with fibrin carrier during arthroscopic rotator cuff repair. Similar to studies that used BMSCs, there were no clinical differences after a follow-up period of 28 months, but a lower recurrent defect rate (14.3%) was observed in the ADSC injection group compared to the control group (28.5%) [122]. In addition to ADSCs, synovial MSCs derived from the subacromial bursa [123] have received recent interest due to their high concentrations in synovial tissue and increased chondrogenic potential than BMSCs [124,125]. Another novel source of stem/progenitor cells for clinically augmentation of rotator cuff healing are urine-derived stem/progenitor cells (USCs). USCs originate from the kidney and can be easily isolated from voided urine or the upper urinary tract with robust proliferation and multilineage differentiation ability [126,127]. The main benefit of USCs is that the harvesting method typically avoids invasive and painful surgical procedures [126,127]. In a canine model of infraspinatus tendon insertion detachment and repair, autogenous USC sheet implantation significantly increased the bone volume/total volume and trabecular thickness, and the USC sheet group exhibited increased fibrocartilage coverage, additional proteoglycan deposition, and higher collagen birefringence together with higher failure load and stiffness compared to the control group [114]. Together, these studies show that use of adult-derived stem/progenitor cells is promising for enhancing rotator cuff healing and regeneration.

In addition to the adult stem cells, umbilical cord blood-derived MSCs (UCB-MSCs) are promising alternatives for clinical-scale allogeneic transplantation as they have high differential potential and can be easily collected and expanded post-natally [128,129]. Relative to BMSCs and ADSCs, they could be cultured the longest and showed the highest proliferation capacity [130]. Pre-clinical studies showed therapeutic effects of UCB-MSCs on tendon repair after rotator cuff injury. For example, ultrasound-guided injection of UCB-MSCs with polydeoxyribonucleotide in a chronic rabbit model of full-thickness rotator cuff tendon tear showed histologic improvements in terms of newly regenerated collagen type 1 fibers, cell proliferation activity and angiogenesis as well as gait analysis in terms of walking distance, fast walking time, and mean walking speed compared to the other groups [112]. However, another clinical trial of UCB-MSCs on ACL reconstruction in a 2-years follow-up showed no treatment-related adverse events but also no improvement in enhancing tendon graft-mediated healing compared to the control groups [131]. Thus, future clinical studies are required to verify the efficacy of UCB-MSCs.

While there have been promising indications for using MSC-based therapy to enhance rotator cuff healing, these clinical studies are limited in number, likely due to the high risks associated with the stem/progenitor cell-based therapies, including invasive harvesting method, possible tumorigenic growth, administration site reactions, and other adverse effects. Also, the complexity of such procedures coupled with increased treatment cost may pose a barrier to patient recruitment and participation. Thus, further preclinical studies are needed to demonstrate the cost-effectiveness of stem/progenitor cell-mediated repair before widespread clinical application can become a reality. In addition, application of stem/progenitor cells could be combined with bio-mimic or bio-inductive scaffolds to achieve greater therapeutic effect on multi-tissue regeneration. A recent study demonstrated that a bioactive collagen scaffold supported tendon-derived cell growth and when used in a prospective clinical trial, resulted in improved patient outcome in terms of shoulder function, quality of life, and pain reduction, highlighting the feasibility and safety for use in rotator cuff augmentation [108]. Combining such scaffolds with stem/progenitor cells may enhance their efficacy further.

#### 5.3.2 Application of growth factors

As an alternative to stem/progenitor cells, there has been growing scientific interest in using exogenously supplied growth factors or *in situ* delivery of stimulated endogenous growth factors to improve rotator cuff regeneration [[132], [133], [134]]. Single growth factor administration, including vascular endothelial growth factor (VEGF), insulin-like growth factor-1 (IGF-1), fibroblast growth factor-2 (FGF-2), transforming growth factor-ß (TGF-ß), and platelet-derived growth factor (PDGF), have been shown to promote tendon healing through different signaling pathways for *in vitro* and *in vivo* experiments. These studies are comprehensively described and summarized in other excelle nt reviews [135,136]. Presently, there have been several clinical studies assessing the effect of single growth factors for rotator cuff repair such as GDF-7/BMP-12 [137,138]. However, such studies are few, likely due to high cost, short growth factor half-life, and potential off-target side effects [134]. As such, application of low-cost growth factor mixtures such as platelet-rich plasma (PRP) has gained interest. PRP is the platelet-rich fraction of blood, and upon platelet activation, releases copious amounts of growth factors, including IGF-1, PDGF, VEGF, and TGF-ß. Such growth factors have been implicated in tendon healing [132] and thus PRP is a promising candidate for biological augmentation of rotator cuff injury. In addition, PRP is easily prepared and is lower in cost relative to recombinant growth factors. The preparation of PRP involves drawing blood, enrichment of the platelet-containing fraction, and injection into the injury site [104]. Furthermore, since PRP can be obtained autologously, there is little-to-no allergic reactions or major side effects expected [104]. Indeed, *in vitro* and *in vivo* studies utilizing PRP have demonstrated promising results for biological augmentation of rotator cuff repair [[139], [140], [141]]. In contrast, numerous clinical trials involving PRP show conflicting results. For example, a large number of studies failed to show significant effects on healing, recurrent defect rate, pain, or functional outcomes [[142], [143], [144], [145], [146], [147], [148]], whereas other studies showed significantly improved structural outcomes such as decreased recurrent defect rate compared with repairs without PRP augmentation [[149], [150], [151], [152], [153]]. Several factors likely contribute to these conflicting results, such as the method of PRP preparation, timing of treatment and activation (pre- or post-injection), variation in leukocyte content of the PRP, PRP dose and retention at the injury site. As such, there have been additional efforts aimed at optimal preparation of PRP such as separation into leukocyte-rich and leukocyte-poor fractions [154]. In such studies, it has been postulated that leukocyte-rich PRP is highly pro-inflammatory due to the presence of catabolic cytokines, which may antagonize the effects of anabolic growth factors [154], whereas other studies suggest that leukocyte-rich PRP is more beneficial in tendinopathies [155]. In addition, there have been recent attempts to modulate healing efficacy by combining PRP with ADSCs [156]. Besides exogenously supplied growth factors, another technically-easy and cost-effective method for delivering growth factors is the microfracture technique, which results in a “super clot” containing a combination of growth factors, platelets, MSCs, and other vascular elements [134]. When the microfracture method was used in combination with hyaluronic acid injection for surgical repair of rotator cuff tear in a rabbit model, improved bone-tendon healing was observed with increased chondrocytes and ECM production as well as increased biomechanical strength at the repair site 12 weeks post-surgery [133]. Thus, application of single growth factors or crude mixtures (via PRP preparation or microfracture surgical technique) represent potent means for improving rotator cuff healing and strength.

#### 5.3.3 Application of exosomes

In addition to stem/progenitor cells and growth factors, another promising alternative is exosome-based therapy, which has been demonstrated to exert potent regenerative effects for rotator cuff healing. Exosomes are nano-sized (30–150 ​nm in diameter) extracellular vesicles naturally secreted by various cell types including cancer cells, endothelial cells, immune cells, and also MSCs from different sources such as bone marrow, adipose and human umbilical cord, etc. [157]. The most attractive feature of exosomes is that they are cell-free and regarded as less complex from a regulatory standpoint, with clinical trials underway following emergency use authorization from the United States Food and Drug Administration. The lipid bilayer structure of exosomes enable the transfer of messages encoded in protein, lipids, mRNA, miRNA, and metabolites, which facilitate intracellular communication [158,159]. Compared with MSCs, MSC-derived exosomes show similar biological activity such as anti-inflammatory, pro-regenerative and anti-apoptotic effects, however, they are more stable, easier to produce and have limited immune rejection [160,161]. As such, the therapeutic effect and mechanism of exosomes during tissue healing have attracted increased interest for enhancing rotator cuff repair.

In this respect, there are several *in vitro* and *in vivo* studies that highlight the potential of exosome-based therapies. Preclinical *in vitro* studies have shown that BMSC-derived exosomes containing TGF-β1 promoted proliferation, migration, and fibrotic activity in rotator cuff tenocytes, which may enhance rotator cuff tendon healing [162]. Similarly, circulating exosomes were also reported to improve tenocyte proliferation and migration by upregulating tenogenic markers [163]. *In vivo* studies also showed that in a mouse bone-tendon reconstruction model, local administration of hydrogel-delivered, BMSC-derived exosomes increased the number of M2 macrophages as well as anti-inflammatory and chondrogenic-related factors, while decreasing the M1 macrophages and related proinflammatory factors [118]. This immunomodulatory response led to increased cell proliferation and decreased cell apoptosis at early timepoints with the hydrogel-delivered, BMSC-derived exosome group exhibiting increased fibrocartilage formation and improved biomechanical properties including maximum force, strength, and elastic modulus at 1 month post-surgery [118]. Together, these findings provide a basis for the potential clinical use of exosomes in rotator cuff tendon repair.

In addition to the regenerative effects on tendon, exosomes have been demonstrated to prevent atrophy, fatty degeneration, inflammation, and vascularization of the muscles in massive or chronic rotator cuff tears. Wang *et al.* demonstrated that in a rat model of massive rotator cuff tear (detachment of both supraspinatus and infraspinatus tendons), injection of ADSCs-derived exosomes (into the proximal and distal side of the supraspinatus muscle) led to decreased loss of supraspinatus muscle weight, reduced adipose content as well as lower macrophage density and vascularization than the saline group at 8 and 16 weeks [164]. In contrast, myofiber regeneration and biomechanical properties were significantly elevated compared with those in the saline-treated group [164]. In addition, the effect of ADSCs-derived exosomes on fatty degeneration and on tendon-bone healing was further examined in a chronic rabbit model of rotator cuff tear [165]. Local injection of ADSC-derived exosomes into torn supraspinatus muscle during repair reduced the percentage of fatty degeneration compared to the saline group, promoted bone-tendon tissue healing as evidenced by increased fibrocartilage regeneration, abundant collagen II and tenascin-C expression, and a more homogenous healing response that included less fibrotic tissue, as well as improved biomechanical properties [165]. Together, these results suggest that ADSC-derived exosomes exhibit great regenerative potential as a cell-free adjunctive therapy to inhibit muscle fatty degeneration and improve rotator cuff healing [165]. In the future, additional clinical trials are required to elucidate the effects of exosomes and their therapeutic effects on rotator cuff healing.

#### 5.3.4 Regulatory environment for stem/progenitor cell, growth factor, and exosome applications

To ensure successful clinical applications of stem/progenitor cells, growth factors, and exosomes, a conducive regulatory environment is needed. Specifically, regulatory requirements must maintain a judicious balance between ensuring adequate innovation to make safe and effective therapies available as well as adequate compliance to avoid unscientific interventions with broad and ambiguous claims [166]. In this regard, established regulatory frameworks and guidance documents from the United States (Human cells, tissues, and cellular and tissue-based products HCT/Ps, 21 CFR part 1271) [167] and European Union (Commission Directive 2004/23/EC and 2006/17/EC) [168] regulatory frameworks have been used to inform the development of acceptable criteria for MSC-based products in Asian markets such as India, Japan, Korean, and Singapore [169]. While regulations differ among different nations, the central principle is the same – the demonstration of safety and efficacy via standardized testing and procedures [170,171]. This includes steps to ensure that: (i) the donor poses minimal risk of transmitting infectious or genetic disease; (ii) the manufacturing and processing steps pose low risk of contamination or damage; (iii) the cell type(s) as well as their purity and potency are known; and (iv) the product will be safe and effective after transplantation to a recipient [170,171]. For example, in the United States, a HCT/P is exempted from specific governmental regulations only if the cell is both harvested and used in the same surgical procedure. Other exemptions may also consider whether a HCT/P is minimally manipulated (i.e., processing does not alter the original relevant characteristics of a structural tissue or relevant biological characteristics of cells or tissues) and whether it is for homologous use (i.e., the HCT/P performs the same basic function(s) in the recipient as in the donor) [172]. In particular, the regulatory environment for combination products such as those involving biomaterials, stem cells, and cell-derived products such as growth factor or exosomes is highly relevant and must be well understood by regenerative medicine practitioners and clinicians. This topic is well reviewed by Tian *et al.* with respect to bioactive, combinatorial products [173] as well as Woods and MacLoughlin with respect to exosome-based products [174]. As such, the regulatory environment is a crucial aspect that will guide the development of future stem/progenitor cell, growth factor, and exosome applications.

#### 5.3.5 Future trends for stem/progenitor cell, growth factor, and exosome applications

In summary, there have been significant research advances for stem/progenitor cell-, growth factor, and exosome-mediated rotator cuff regeneration. These include novel applications of MSCs such as BMSCs, ADSCs, USCs, and UCB-MSCs, recombinant growth factors such as GDF-7/BMP-12, and biologics that either contain growth factor mixtures such as PRP or cell-free exosomes. While varying degrees of success have been reported in such research, it is noteworthy that few (if any) of these therapies are explicitly targeted for bone-tendon-muscle multi-tissue regeneration. Future efforts such as improved formulations and optimal dosages of PRP, and novel combinations of stem/progenitor cells, growth factors, and exosomes are expected to enhance rotator cuff multi-tissue regeneration. However, these advances will need to comply with existing healthcare regulatory rules to ensure safety and effectiveness.

### 5.4 Perspective: targeting of multi-tissue, musculoskeletal units

Given the multi-faceted challenges of rotator cuff repair, including incomplete healing, scar tissue formation, musculoskeletal tissue retraction and degeneration, and the high interdependency of musculoskeletal tissues in terms of maintaining a structure–function relationship for unhindered movement, a single-pronged approach such as targeting the site of rotator cuff tears alone (i.e., at the bone-tendon interface) may not suffice for restoring movement, particularly for large-to-massive injuries. While there have been unprecedented advances in surgical techniques, development of medical devices, and novel applications of bioregenerative cues such as stem/progenitor cells, exosomes, and growth factors, there have been few efforts directed at addressing multi-tissue degeneration within the bone-tendon-muscle unit comprehensively. As such, we propose that functional tissue engineering and regenerative medicine efforts employ a strategy to target and engineer multiple tissues which comprise a single musculoskeletal unit.

In support of this, there have been a large number of reports on the development and application of multi-tissue grafts. Such preclinical and clinical studies include the use of bone-tendon allografts [175] and semitendinosus and gracilis free muscle-tendon autografts [176] for treating musculoskeletal injuries, including chronic rotator cuff tears. Indeed, it has been noted that treating rotator cuff tears is not merely about addressing tendon injuries [177]. This is because bone loss typically accompanies rotator cuff injury [59], and treating such pathology via subcutaneous injections of sclerostin antibodies resulted in improved bone mineral density, enhanced tissue biomechanical properties, and excellent bone-tendon integration in an acute rat rotator cuff resection model [178]. In addition, bioprinting of growth factors makes it possible to spatially control osteoblast, tenocyte and myocyte differentiation simultaneously with a single stem cell population to generate a primitive bone-tendon-muscle unit [179]. Such growth factor biopatterning technology can be combined with various biomaterials such as aligned sub-micron fibrous scaffolds, which are morphologically similar to musculoskeletal ECM, and result in simultaneous spatial control of muscle progenitor cell differentiation and alignment [180]. In addition, novel polymer-based biomaterials that possesses phototunable bone- and tendon-like biomechanical properties such as QHM polyurethane has been developed for reducing stress concentrations and material failure at the interface of tissues with dissimilar mechanical attributes for rotator cuff repair [181]. Furthermore, Wang *et al.* demonstrated that combination of this mechanically-graded, bone- and tendon-like polymer with selectively biopatterned growth factors cues (osteogenic bone morphogenetic protein-2; BMP-2 and tenogenic fibroblast growth factor-2; FGF-2) could influence musculoskeletal differentiation with substrates of increased stiffness favouring osteoblast differentiation and substrates of decreased stiffness favouring tenocyte differentiation *in vitro* [182]. Subsequent *in vivo* studies also showed spatially control of ectopic bone-and tendon-like tissue formation in mouse subcutaneous studies [182]. In addition to developing scaffolds that enable smooth interfacing of tissues with dissimilar properties, scaffolds that automatically change their structure or mechanical properties to adapt to the requirement of the surrounding environment will provide new directions for tissue engineering. You *et al.* synthesized pH-sensitive scaffolds that swelled in response to a decrease in pH and demonstrated that the responsive scaffold could encourage wound healing by inducing a pro-healing gene expression profile indicative of enhanced angiogenesis, granulation tissue formation, and tissue remodeling [183]. Future combination of stem/progenitor cells or growth factors with the responsive scaffold may provide new ideas for advanced therapeutic strategies. Similarly, muscle-tendon autografts [176] and clinical techniques that surgically advance muscle to reduce rotator cuff tension [184] have been used to address issues of fatty muscle degeneration and retraction [58,[185], [186], [187]] to repair massive rotator cuff injuries in the clinic. Special effort has also been made towards deconstructing the cell–matrix interaction in the muscle-tendon junction. Gaffney *et al.* used tissue-specific extracellular matrix derived from muscle and tendon to determine how cells of each tissue interact with the microenvironment of the opposing tissue, aiming to inform future methods to engineer a more relevant muscle-tendon unit [188]. Together, these studies support the idea that engineering a bone-tendon-muscle unit will be clinically impactful for the treatment of rotator cuff tears, which raises the question of where such musculoskeletal units may be obtained from.

To practically attain musculoskeletal multi-tissue units for implementation in a clinical setting, numerous engineering and biological advances are needed. For example, technical innovations in biomaterial fabrication and maturation are required to develop grafts or tissue engineered constructs capable of biomimicking bone, tendon, and muscle tissues as well as their respective interfaces [2]. These include approximation of mechanical and biological attributes of the bone-tendon-muscle unit such as tensile properties and stress-reducing features, as well as native tissue structure and composition, respectively, while simultaneously addressing clinical concerns such as avoiding suture tearing through the tendon or premature loss of surgical repair construct. This can be accomplished through careful balancing of biomaterial attributes including scaffold architecture for eventual host cell colonization, choice of material and fabrication approach to attain desired mechanical properties, and expected degradation rate to sustain repair integrity during wound healing, which are reviewed in additional detail by Wang et al. [2] Also, improved understanding of the biology of musculoskeletal tissue development and maturation as well as the wound healing response is vital to ensure that such bone-tendon-muscle grafts or tissue engineered constructs can be integrated well with host tissue under relevant rotator cuff injury pathophysiological conditions [3]. These include employing appropriate biological cues that direct musculoskeletal tissue formation, maturation, and multi-tissue integration as well as those that govern wound healing [3]. In particular, the use of appropriate growth factors, ECM, and mechanical forces on orchestrating musculoskeletal differentiation as well as positive modulation of inflammation for musculoskeletal tissue healing, integration of clinical grafts and devices, and vascularization can be gleaned from developmental and wound healing studies, which are reviewed in additional detail by Xu et al. [3] Thus, engineering innovations and biological advances are both needed to play crucial roles in the development and application of bone-tendon-muscle multi-tissue units for rotator cuff repair.

In summary, rotator cuff tears are not simply confined to the location of the injury site. Degenerative changes to associated tissues such as bone, tendon, and muscle disrupt the structure and function of the shoulder, exacerbating the clinical condition. Although various non-surgical management methods, surgical techniques, and commercially available biomaterials exist, tissue engineering and regenerative medicine approaches that focus on engineering a bone-tendon-muscle unit to replenish injured tissue, facilitate secure tissue attachment, and restore normal shoulder kinematics (e.g., biomechanical strength, tension, degree of shoulder rotation) may offer a way to address unmet clinical challenges in rotator cuff tears. Fabricating such constructs will require drawing inspiration from domain knowledge in musculoskeletal development and integration, maturation, and wound healing to specify multiple cell fates within a single multi-tissue unit.

## 6 Conclusion

Rotator cuff tears are highly prevalent and can be detrimental to laborers and athletes, posing great socioeconomic burden to numerous countries. Detailed anatomical examination shows a highly integrated structure–function relationship in which the rotator cuff exists as a unified complex of four bone-tendon-muscle units. Indeed, rotator cuff injury can cause bone loss, tendon retraction and fibrosis, as well as muscle fatty degeneration through loss of musculoskeletal tension. Current clinical state-of-the-art includes non-surgical management such as physical rehabilitation and surgical management such as anatomical (Open repair, mini-open repair, and arthroscopic repair) and non-anatomical (RTSA, tendon transfer, and ASCR), which involve use of natural or synthetic tendon grafts and bone fixation devices. Experimental trials involving use of stem cells and/or growth factors have been conducted but various regulatory hurdles must be addressed along with multi-disciplinary integration of biological, engineering, and clinical knowledge to develop advanced biological and tissue engineering strategies to treat bone, tendon, and muscle pathologies associated with rotator cuff tears. Continued communication and collaboration among life scientists, engineers, and clinicians will facilitate the rational formulation and implementation of functional tissue engineering approaches for multi-tissue repair and regeneration.

## Authorship

Category 1

Conception and design of study: X. Zhang, D. Wang, D.F.E. Ker.

Category 2

Drafting the manuscript: X. Zhang, D. Wang.

revising the manuscript critically for important intellectual content: Z. Wang, S.K.K. Ling, P.S.H. Yung, R.S. Tuan, D.F.E. Ker.

Category 3

Approval of the version of the manuscript to be published (the names of all authors must be listed): X. Zhang, D. Wang Z. Wang, S.K.K. Ling, P.S.H. Yung, R.S. Tuan, D.F.E. Ker.

X. Zhang: Writing - Original Draft; D. Wang: Writing - Original Draft; Z. Wang: Writing - Review & Editing; S.K.K. Ling: Writing - Review & Editing; P.S.H. Yung: Writing - Review & Editing; R.S. Tuan: Writing - Review & Editing; D.F.E. Ker: Supervision, Writing - Review & Editing.

## Declaration of competing interest

The authors have no conflict of interest relevant to this review.
